# Supplementary material for: A Multicenter Clinical Diagnostic Accuracy Study of SureStatus, an Affordable, WHO Emergency Use-Listed, Rapid, Point-Of-Care Antigen-Detecting Diagnostic Test for SARS-CoV-2
Source: Microbiol Spectr. 2022 Sep 6;10(5):e01229-22. doi: 10.1128/spectrum.01229-22 (PMC9604065; doi:10.1128/spectrum.01229-22)
Supplement: Supplemental file 1 — Supplemental material. Download spectrum.01229-22-s0001.pdf, PDF file, 1.0 MB [file spectrum.01229-22-s0001.pdf]

Supplementary material

Table of content

Table: S1 Study Group 2

Table: S2 Participant Questionnaire: Symptoms attributed to possible COVID-19 3

Questionnaire: S3 System Usability Scale (SUS) 5

Questionnaire: S4 Ease-of-Use Assessment (EoU) 6

Figure: S5 Matrix for Ease-of-Use Assessment 15

Table: S6 Temperature and humidity across study sites 17

Table: S1 Study Group

Department of Public Health Rhein Neckar Region, Heidelberg, Germany	Dr. Kholoud Assaad
	Dr. Andrea Fuhs
	Christopher Harter
	Cristopher Schulze
	Gunter Schmitt
Division of Infectious Disease and Tropical Medicine, Heidelberg University Hospital, Heidelberg, Germany	Martina Fink
	Maximilian Schirmer
	Annika Small
	Matthias Meinlschmidt
	Valerie Dürr
	Alina Schuckert
	Salome Steinke
	Henrik Ellinghaus
	Alexander Penning
	Loai Abutaima
Institute of Tropical Medicine and International Health, Charité – Universitätsmedizin Berlin, Berlin, Germany	Mandy Kollatzsch
	Mia Wintel
	Franka Kausch
	Franziska Hommes
	Alisa Bölke
	Julian Bernhard
	Claudia Hülso
	Elisabeth Linzbach
Medical Directorate, Charité – Universitätsmedizin Berlin, Berlin, Germany	Heike Rössig
Institute of Tropical Medicine and International Health, Charité – Universitätsmedizin Berlin, Berlin, Germany	Maximilian Gertler
Charité Comprehensive Cancer Center, Charité – Universitätsmedizin Berlin, Berlin, Germany	Susen Burock
Department of Pediatric Surgery, Charité – Universitätsmedizin Berlin, Berlin, Germany	Katja von dem Busche
Berlin Institute for Clinical Teratology and Drug Risk Assessment in Pregnancy, Institute of Clinical Pharmacology and Toxicology, Charité – Universitätsmedizin Berlin, Berlin, Germany	Stephanie Patberg

Table: S2 Participant Questionnaire: Symptoms attributed to possible COVID-19

Did you have any symptoms of possible COVID-19 on the day of the test?	○ No<br>○ Yes
Increased temperature / fever?	○ No<br>○ Yes
Did you measure your fever?	○ No<br>○ Yes
Highest temperature (in Celsius)	*(Free text)*
Cough	○ No<br>○ Yes
Do you have a productive cough?	○ No<br>○ Yes
Sore throat	○ No<br>○ Yes
Shortness of breath	○ No<br>○ Yes
Muscle pain / Body aches	○ No<br>○ Yes
Fatigue	○ No<br>○ Yes
Headache	○ No<br>○ Yes
Runny nose	○ No<br>○ Yes
Chest pain	○ No<br>○ Yes
Diarrhea	○ No<br>○ Yes
Nausea / vomiting	○ No<br>○ Yes
Loss of taste or smell	○ No<br>○ Yes
Other	○ No<br>○ Yes

If yes, please specify	*(Free text)*
The earliest onset of symptoms attributed to possible COVID-19	*(Day / Month / Year)*

Questionnaire: S3 System Usability Scale (SUS)

"Evaluation of the performance of novel rapid diagnostics for SARS-CoV-2 at point-of-care"

System Usability Scale (SUS)

© Digital Equipment Corporation, 1986 adapted format, version 1.0: 02/05/20

Name of the test: ____

User identifier and study site: __________________________ Date: ________________________

	Strongly disagree				Strongly agree
1. I think that I would like to use this system frequently	1	2	3	4	5
2. I found the system unnecessarily complex	1	2	3	4	5
3. I thought the system was easy to use	1	2	3	4	5
4. I think that I would need the support of a technical person to be able to use this system	1	2	3	4	5
5. I found the various functions in this system were well integrated	1	2	3	4	5
6. I thought there was too much inconsistency in this system	1	2	3	4	5
7. I would imagine that most people would learn to use this system very quickly	1	2	3	4	5
8. I found the system very cumbersome to use	1	2	3	4	5
9. I felt very confident using the system	1	2	3	4	5
10. I needed to learn a lot of things before I could get going with this system	1	2	3	4	5

Using SUS

The SU scale is generally used after the respondent has had an opportunity to use the system being evaluated, but before any debriefing or discussion takes place. Respondents should be asked to record their immediate response to each item, rather than thinking about items for a long time.

All items should be checked. If a respondent feels that they cannot respond to a particular item, they should mark the center point of the scale.

Questionnaire: S4 Ease-of-Use Assessment (EoU)

"Evaluation of the performance of novel rapid diagnostics for SARS-CoV-2 at point-of-care"

Thank you for your time to answer this questionnaire (about 20 minutes).

Your input is very valuable!

OVERALL QUESTIONS

1. **User identifier**
2. **Date of filling the questionnaire**
3. **In which country do you currently work?**
4. **At which facility / study site do you currently work?**

Mark only one oval.

- Reilingen/ Schwetzingen (Heidelberg)
- Berlin
- Liverpool
- Macae CTC
- Marica (Guapi)
- UFRJ

5. **Which test are you assessing?**

Mark only one oval.

- Rapigen
- Standard F
- Standard Q
- Bioeasy
- Fujirebio
- Bionote
- Mologic

6. **About how many times did you perform this test approximately?**

Mark only one oval.

- only observed use
- < 10
- 10-100
- > 100

7. About how many times did you observe the use of this test (not performed yourself)?

Mark only one oval.

- < 10
- 10 - 50
- > 50-100
- > 100

8. What is your profession?

9. How many years of laboratory experience do you have?

10. How many years of working experience in limited resource settings do you have?

11. How much experience do you have with interpreting the results of lateral flow tests or rapid diagnostics (e.g., for HIV, malaria, pregnancy)?

Please note that we refer here to your experience with INTERPRETING the test results. If you do not conduct the test yourself, but do inform patients about the test results, we also consider that as experience with INTERPRETING the test results.

Mark only one oval.

- None
- < 1 year
- 1 – 3 years
- > 3 years

TEST SPECIFIC QUESTIONS

TRAINING

12. How satisfied were you with the following components of the test training?

Mark only one oval per row.

	Very satisfied	Satisfied	Neither	Dissatisfied	Very dissatisfied
Instructions for Use					
Standard Operating Procedures					
Face to face demonstration					

13. **What additional materials (if any) do you think should be provided as part of the training?**

- None
- Other: ____________________

14. How long should be the training of this test?

Mark only one oval.

- Self-explanatory, no need
- for training 1-2 hours
- 2-4 hours
- Half a day
- Full day

15. Do you consider proficiency testing necessary?

Proficiency testing as in assessing the user's performance or ability to run the test following the training.

Mark only one oval.

- Y
- N
- Other:

16. Please comment here on the need for proficiency testing

17. After how many of these tests do you feel you could perform the test on your

own (having access to the training material)?

Mark only one oval.

- 1-2 tests
- 3-5 tests
- 6-10 tests
- >10 tests

<u>*ASSESSMENT OF TEST*</u>

18. How satisfied are you with the quality of each of the components in the kit (in terms of ease-of-use and fit for purpose)?

Mark only one oval per row

	Very satisfied	Satisfied	Neither	Dissatisfied	Very dissatisfied
External paper box of kit	⬭	⬭	⬭	⬭	⬭
Assay diluent tube	⬭	⬭	⬭	⬭	⬭
Filter cap (if necessary)	⬭	⬭	⬭	⬭	⬭
Buffer bottle (if necessary)	⬭	⬭	⬭	⬭	⬭
Swab for specimen collection	⬭	⬭	⬭	⬭	⬭
Test cartridge / device	⬭	⬭	⬭	⬭	⬭
Test cartridge packing / pouch	⬭	⬭	⬭	⬭	⬭
Reader (if necessary)	⬭	⬭	⬭	⬭	⬭

19. Which kit component(s) should be improved in your opinion (if any)?
Please specify why and how

20. Overall, how satisfied are you with the kit components?
Mark only one oval.

	1	2	3	4	5	
Very satisfied	⬭	⬭	⬭	⬭	⬭	Very dissatisfied

21. How satisfied are you with the overall design of the device in terms of the following features?
Mark only one oval per row.

	Very satisfied	Satisfied	Neither	Dissatisfied	Very dissatisfied
Size of cartridge	⬭	⬭	⬭	⬭	⬭
Space for labeling on the front	⬭	⬭	⬭	⬭	⬭
Size of the well to add sample mix	⬭	⬭	⬭	⬭	⬭
Size of reading window	⬭	⬭	⬭	⬭	⬭
Logical sequence of steps	⬭	⬭	⬭	⬭	⬭

22. Overall, how satisfied are you with the design of the device?
Mark only one oval.

	1	2	3	4	5	
Very satisfied	⬭	⬭	⬭	⬭	⬭	Very dissatisfied

23. Please assess the Test's storage conditions
Mark only one oval.

> 12 months	12 to 6 months	5 to 3 months	< 3 months

Stability of test				
Stability of control material (if appl.)				
	2 – 40°	15 – 35°	15 – 30°	20 – 25°
Storage temperature				

24. Overall, how satisfied are you with the test's storage conditions?

Mark only one oval.

	1	2	3	4	5	
Very satisfied						Very dissatisfied

25. Which part(s) of the device could be improved in your opinion (if any)?

Please specify why and how.

26. Please determine the difficulty of the following steps:

Please consider your day-to-day/routine workload (or that of the people in the lab/area where this test could be implemented) to answer this question.

Mark only one oval per row.

	Very easy	Easy	Neither	Difficult	Very difficult
a) Check expiry date					
b) Remove the test cartridge from the pouch					
c) Label the test cartridge with patient identifier					
d) Label the assay diluent tube with patient identifier					
e) Open the assay diluent tube by removing the seal (if appl.)					
f) Transfer of buffer into diluent tube (if applicable)					
g) Insert the swab into the tube					
h) Ease of swab extraction procedure					
i) Ability to perform swab extraction procedure consistently					
j) Ability to maintain cleanliness of ancillary devices (e.g. pipette) in order to avoid cross contamination					
k) Ease of transferring sample onto device					
l) Ease of transferring exact quantity into the sample well					

m) Trouble shooting	⬭	⬭	⬭	⬭	⬭

27. How satisfied are you with the logical sequence of steps?
Mark only only one oval.

	1	2	3	4	5	
Very satisfied	⬭	⬭	⬭	⬭	⬭	Very dissatisfied

28. Overall, how difficult did you find the steps?
Mark only one oval.

	1	2	3	4	5	
Very easy	⬭	⬭	⬭	⬭	⬭	Very difficult

29. Please assess the time relevant components of the test.
Mark only one oval per row.

	≤ 2 min	3 to 5 min	6 to 10 min	> 10 min
Pre analytic time	⬭	⬭	⬭	⬭
Analytic time	⬭	⬭	⬭	⬭

30. In your opinion, about how many patients could be tested with this test in an 8-hour day?
Mark only one oval per row.

- ⬭ < 10
- ⬭ 10 - 50
- ⬭ > 50-100
- ⬭ > 100

31. Please comment here if you see any potential issues or room for improvement.

READ-OUT of TEST

32. How did you find the results read-out in the following areas?
Mark only one oval per row.

	Very easy	Easy	Neither	Difficult	Very difficult
a) Visibility of the control (C) and in contrast with the background (if applicable)?	⬭	⬭	⬭	⬭	⬭
b) Visibility of the test (T) band in contrast with the background (if applicable)?	⬭	⬭	⬭	⬭	⬭
c) Read-out from Reader (if applicable)	⬭	⬭	⬭	⬭	⬭

d) Interpretation of the test result ⬭ ⬭ ⬭ ⬭ ⬭

33. Do you foresee any issues with reading these results considering the lighting conditions in the settings you currently work or have experience with?

Mark only one oval.

- Yes
- No

If yes, please explain here

34. For visual readout: Was there any color on the background of the test result (T) or control band (C) that made the interpretation of the bands difficult?

Mark only one oval.

- Not applicable
- Yes
- No

If yes, which background color was present?

35. How satisfied are you with the reader if applicable (in terms of ease of use and fit for purpose)?

- Not applicable

If applicable, mark only one oval.

	1	2	3	4	5	
Very satisfied	⬭	⬭	⬭	⬭	⬭	Very dissatisfied

36. Overall, how satisfied are you with the reader (if applicable)?

- Not applicable

If applicable, mark only one oval.

	1	2	3	4	5	
Very satisfied	⬭	⬭	⬭	⬭	⬭	Very dissatisfied

37. Which component(s) of the reader (if applicable) should be improved in your opinion (if any)?

Please specify why and how

OVERALL ASSESMMENT

38. Overall, how did you find the use of this rapid COVID-19 diagnostic tool:
Mark only one oval.

	1	2	3	4	5	
Very easy	⬭	⬭	⬭	⬭	⬭	Very difficult

39. Please comment here on the use:

40. Which option(s) do you consider feasible in your setting?
Tick all that apply.

- ☐ Sequential testing (run tests one by one)
- ☐ Batch testing (run multiple tests at the time)

41. **Which aspect(s) of this test could cause difficulties in its day-to-day use?** *Tick all that apply.*

- ☐ Hands-on time
- ☐ Total assay time to result
- ☐ Batch processing
- ☐ Throughput
- ☐ Test results interpretation
- ☐ Overall number of steps
- ☐ Time-sensitive steps
- ☐ Cartridge design
- ☐ Quality of material
- ☐ Training requirements
- ☐ Storage conditions and stability
- ☐ Waste management requirements
- ☐ I don't know
- ☐ None, I see no barriers for implementation

42. Please give a short explanation for each of the aspects you selected above e.g. what could be the challenges in the day-to-day use:

SETTINGS UF USE

43. Do you see this test being used in its current form in your setting in your country?
Mark only one oval.

- ⬭ Yes
- ⬭ No
- ⬭ I don't know

Please elaborate:

---

44. If yes, at which health care level(s) do you see this test being implemented in your country

Tick all that apply.

- ☐ Family doctor/General physician
- ☐ Peripheral hospital/lab
- ☐ Reference hospital/lab
- ☐ At a testing site operated by trained staff without specific laboratory expertise

45. If you don't see this test being used in its current form, which aspects should be changed to make it suitable for use in your setting in your country:

46. Do you see this test being used in its current form in your setting in low- and middle-income countries?

- ○ Yes
- ○ No
- ○ I don't know

Please elaborate:

47. At which health care level(s) do you see this test being implemented in low- and middle-income countries?

- ☐ Family doctor/General physician
- ☐ Primary health care
- ☐ Health Centre/microscopy lab
- ☐ District hospital/lab
- ☐ Reference hospital/lab
- ☐ Other:
- ☐ I cannot answer this question as I have no work experience in those countries

48. If you don't see this test being used in its current form, which aspects should be changed to make it suitable for use in your setting in low-and middle-income countries?

49. Anything else you would like to add?

THANK YOU VERY MUCH!

Figure: S5 Matrix for Ease-of-Use Assessment

#	Question	green	green	green	yellow	yellow	amber	amber
1	User Identifier (first name, surname)	-	-	-	-	-	-	-
2	Date of filling the questionnaire	-	-	-	-	-	-	-
3	In which country do you currently work?	-	-	-	-	-	-	-
4	At which facility / study site do you currently work?	-	-	-	-	-	-	-
5	Which test are you assessing?	-	-	-	-	-	-	-
6	About how many times did you perform this test approximately?	-	-	-	-	-	-	-
7	About how many times did you observe the use of this test (not performed yourself)?	-	-	-	-	-	-	-
8	What is your profession?	-	-	-	-	-	-	-
9	How many years of laboratory experience do you have?	-	-	-	-	-	-	-
10	How many years of working experience in limited resource settings do you have?	-	-	-	-	-	-	-
11	How much experience do you have with interpreting the results of lateral flow tests or rapid diagnostics (e.g. for HIV, malaria, pregnancy)?	-	-	-	-	-	-	-
12	How satisfied were you with the following components of the test training? [Instructions for use]	very satisfied	satisfied	-	neither	-	dissatisfied	very dissatisfied
12	How satisfied were you with the following components of the test training? [Standard operating procedures]	-	-	-	-	-	-	-
12	How satisfied were you with the following components of the test training? [Face - to - face demonstration]	-	-	-	-	-	-	-
13	What additional materials (if any) do you think should be provided as part of the training?	None	-	-	1-2	-	>2	-
14	How long should the training of this test be?	Self - explanatory, no n	1 - 2 hours of training	2 - 4 hours	Half a day	-	Full Day	-
15	Do you consider proficiency testing necessary?	No	-	-	-	-	Yes	-
16	Please comment here on the need for proficiency testing:	-	-	-	-	-	-	-
17	After how many of these tests do you feel you could perform the test on your own?	1 - 2 tests	-	-	3 - 5 tests	-	6 - 10 tests	10 tests
18	How satisfied are you with the quality of each of the components in the kit (in terms of ease of use and fit for purpose)? [External paper box of the kit]	very satisfied	satisfied	-	neither	-	dissatisfied	very dissatisfied
18	How satisfied are you with the quality of each of the components in the kit (in terms of ease of use and fit for purpose)? [Assay diluent tube]	very satisfied	satisfied	-	neither	-	dissatisfied	very dissatisfied
18	How satisfied are you with the quality of each of the components in the kit (in terms of ease of use and fit for purpose)? [Filter cap (if necessary)]	very satisfied	satisfied	-	neither	-	dissatisfied	very dissatisfied
18	How satisfied are you with the quality of each of the components in the kit (in terms of ease of use and fit for purpose)? [Buffer bottle (if necessary)]	very satisfied	satisfied	-	neither	-	dissatisfied	very dissatisfied
18	How satisfied are you with the quality of each of the components in the kit (in terms of ease of use and fit for purpose)? [Swab for specimen collection]	very satisfied	satisfied	-	neither	-	dissatisfied	very dissatisfied
18	How satisfied are you with the quality of each of the components in the kit (in terms of ease of use and fit for purpose)? [Test cartridge - device]	very satisfied	satisfied	-	neither	-	dissatisfied	very dissatisfied
18	How satisfied are you with the quality of each of the components in the kit (in terms of ease of use and fit for purpose)? [Test cartridge - packing pouch	very satisfied	satisfied	-	neither	-	dissatisfied	very dissatisfied
18	How satisfied are you with the quality of each of the components in the kit (in terms of ease of use and fit for purpose)? [Reader (if necessary)]	very satisfied	satisfied	-	neither	-	dissatisfied	very dissatisfied
19	Which kit component(s) should be improved in your opinion (if any)?	-	-	-	-	-	-	-
20	Overall, how satisfied are you with the kit components?	1	2	-	3	-	4	5
21	How satisfied are you with the design of the following features? [Size of the cartridge]	very satisfied	satisfied	-	neither	-	dissatisfied	very dissatisfied
21	How satisfied are you with the design of the following features? [Space for labeling on the front (e.g. patient ID)]	very satisfied	satisfied	-	neither	-	dissatisfied	very dissatisfied
21	How satisfied are you with the design of the following features? [Size of the well to add sample mix]	very satisfied	satisfied	-	neither	-	dissatisfied	very dissatisfied
21	How satisfied are you with the design of the following features? [Size of reading window]	very satisfied	satisfied	-	neither	-	dissatisfied	very dissatisfied
21	How satisfied are you with the design of the following features? [Logical sequence of steps]	-	-	-	-	-	-	-
22	Overall, how satisfied are you with the design of the device?	1	2	-	3	-	4	5
23	Please assess the test's storage conditions. [Stability of test]	12 months	-	-	12 to 6 months	5 to 3 months	3 months	-
23	Please assess the test's storage conditions. [Stability of control material (if applicable)]	12 months	-	-	12 to 6 months	5 to 3 months	3 months	-
23	Please assess the test's storage conditions. [Storage temperature]	2 - 40°	-	-	15- 35°	15 - 30°	20 - 25°	-
24	Overall, how satisfied are you with the test's storage conditions?	1	2	-	3	-	4	5
25	Which part(s) of the device could be improved in your opinion (if any)?	-	-	-	-	-	-	-
26	Please determine the difficulty of the following steps: [a) Check expiry date]	Very easy	Easy	-	Neither	-	Difficult	Very difficult
26	Please determine the difficulty of the following steps: [b) Remove the test cartridge from the pouch]	Very easy	Easy	-	Neither	-	Difficult	Very difficult
26	Please determine the difficulty of the following steps: [c) Label the test cartridge with the patient identifier]	Very easy	Easy	-	Neither	-	Difficult	Very difficult
26	Please determine the difficulty of the following steps: [d) Label the assay diluent tube with the patient identifier]	Very easy	Easy	-	Neither	-	Difficult	Very difficult
26	Please determine the difficulty of the following steps: [e) Open the assay diluent tube by removing the seal (if appl.)]	Very easy	Easy	-	Neither	-	Difficult	Very difficult
26	Please determine the difficulty of the following steps: [f) Transfer of buffer into diluent tube (if appl.)]	Very easy	Easy	-	Neither	-	Difficult	Very difficult
26	Please determine the difficulty of the following steps: [g) Insert the swab into the tube]	Very easy	Easy	-	Neither	-	Difficult	Very difficult
26	Please determine the difficulty of the following steps: [h) Ease of swab extraction procedure]	Very easy	Easy	-	Neither	-	Difficult	Very difficult
26	Please determine the difficulty of the following steps: [i) Ability to perform extraction procedure consistently]	Very easy	Easy	-	Neither	-	Difficult	Very difficult
26	Please determine the difficulty of the following steps: [j) Ability to maintain cleanliness of ancillary devices (e.g. pipette) in order to avoid cross-contami	Very easy	Easy	-	Neither	-	Difficult	Very difficult
26	Please determine the difficulty of the following steps: [k) Ease of transferring sample onto device]	Very easy	Easy	-	Neither	-	Difficult	Very difficult
26	Please determine the difficulty of the following steps: [l) Ease of transferring exact quantity into the sample well]	Very easy	Easy	-	Neither	-	Difficult	Very difficult
26	Please determine the difficulty of the following steps: [m) Trouble shooting]	Very easy	Easy	-	Neither	-	Difficult	Very difficult

27	How satisfied are you with the logical sequence of steps?	1	2	-	3	-	4	
28	Overall, how difficult did you find the steps?	1	2	-	3	-	4	4
29	Please assess the time relevant components of the test. [Pre-analytic time]	2 min	-	-	3 to 5 min	6 to 10 min	10 min	-
29	Please assess the time relevant components of the test. [Analytic time]	2 min	-	-	3 to 5 min	6 to 10 min	10 min	-
30	In your opinion, about how many patients could be tested with this test in an 8-hour day?	100	-	-	50 - 100	-	10 - 50	10
31	Please comment here if you see any potential issues or room for improvement.	-	-	-	-	-	-	-
32	How did you find the results read-out in the following areas: [a) Visibility of the control (C) band in contrast with the background (if appl.)]	Very easy	Easy	-	Neither	-	Difficult	Very difficult
32	How did you find the results read-out in the following areas: [b) Visibilty of the test (T) band in contrast with the background (if appl.)]	Very easy	Easy	-	Neither	-	Difficult	Very difficult
32	How did you find the results read-out in the following areas: [c) Read-out from Reader (if appl.)]	Very easy	Easy	-	Neither	-	Difficult	Very difficult
32	How did you find the results read-out in the following areas: [d) Interpretation of the test result]	Very easy	Easy	-	Neither	-	Difficult	Very difficult
33	Do you foresee any issues with reading these results considering the lighting conditions in the settings you currently work or have experience with?	-	-	-	-	-	-	-
33	Please elaborate	-	-	-	-	-	-	-
34	For visual readout: Was there any color on the background of the test result (T) or control band (C) that made the interpretation of the bands difficult?	-	-	-	-	-	-	-
34	If yes, which background color was present	-	-	-	-	-	-	-
35	How satisfied are you with the reader (if applicable) in terms of ease of use and fit for purpose?	1	2	-	3	-	4	5
36	Overall, how satisfied are you with the reader (if applicable)?	1	2	-	3	-	4	5
37	Which component(s) of the reader (if applicable) should be improved in your opinion (if any)?	-	-	-	-	-	-	-
38	Overall, how did you find the use of this rapid COVID-19 diagnostic tool?	1	2	-	3	-	4	5
39	Please comment here on the use:	-	-	-	-	-	-	-
40	Which option(s) do you consider feasible in your setting?	Batch testing (run mult	-	-	-	-	Sequential testing (run	-
41	Which aspect(s) of this test could cause difficulties in its day-to-day use?	0	-	-	1	2	3	4
42	Please give a short explanation for each of the aspects you selected above e.g. what could be the challenges in the day-to-day use:	-	-	-	-	-	-	-
43	Do you see this test being used in its current form in your setting in your country?	Yes (please explain below)		-	-	-	No (please explain belo	-
43	Please elaborate	-	-	-	-	-	-	-
44	If yes, at which health care level(s) do you see this test being implemented in your country?	At a testing site operat	Family doctor / Genera	-	Peripheral hospital / la	-	Reference hospital / la	-
45	If you don't see this test being used in its current form, which aspects should be changed to make it suitable for use in your setting in your country:	none	-	-	1-2	-	>2	-
46	Do you see this test being used in its current form in your setting in LOW and MIDDLE INCOME COUNTRIES?	Yes (please explain belo	-	-	-	-	No (please explain belo	-
46	Please elaborate	-	-	-	-	-	-	-
47	At which health care level(s) do you see this test being implemented in low and middle income countries?	Primary health care	Famildy doctor / Gener	Health centre / microsc	District hospital / labor	-	Reference hospital / la	-
48	If you don't see this test being used in its current form, which aspects should be changed to make it suitable for use in your setting in low and middle in	none	-	-	1	2	3	4
49	Anything else you would like to add?	-	-	-	-	-	-	-

Table: S6 Temperature and humidity across study sites

The temperature and the humidity were recorded per site and due to different study site characteristics, the testing environment varied. In Germany, an average temperature of 16·3° Celsius was recorded in Heidelberg and 24·2° Celsius in Berlin, with temperatures not decreasing below 15° Celsius. The average humidity was 34·9% in Heidelberg and 19·9% in Berlin.

**Study Site: Heidelberg**		
**Date**	**Temperature** (Start-End)	**Humidity** (Start-End)
26.02.2021	15·9 - 17·5 ° Celsius	34% - 46%
01.03.2021	12·9 - 18·1 ° Celsius	32% - 29%
02.03.2021	14·0 - 18·9 ° Celsius	31% - 30%
03.03.2021	17·2 - 17·6 ° Celsius	35% - 32%
04.03.2021	17·8 - 18·4 ° Celsius	31% - 31%
05·03·2021	15·2 - 17·0 ° Celsius	40% - 35%
08·03·2021	16·8 - 17·1 ° Celsius	17% - 21%
09·03·2021	13·2 - 15·1 ° Celsius	25% - 28%
10·03·2021	16·2 – 18·3 ° Celsius	32% - 35%
11·03·2021	16·7 – 15·2 ° Celsius	34% - 52%
12·03·2021	14·2 - 17·4 ° Celsius	45% - 40%
15·03·2021	13·8 - 15·6 ° Celsius	43% - 45%
16·03·2021	14·9 – 18·2 ° Celsius	39% - 42%
17·03·2021	15·0 – 16·6 ° Celsius	42% - 42%
18·03·2021	14·0 – 15·8 ° Celsius	36% - 29%
19·03·2021	15·0 – 17·5 ° Celsius	37% - 38%
22·03·2021	15·9 – 17·9 ° Celsius	32% - 31%
23·03·2021	16·5 – 16·1 ° Celsius	35% - 34%
24·03·2021	14·4 – 17·2 ° Celsius	35% - 36%
25·03·2021	15·5 – 20·1 ° Celsius	32% - 34%
**Average**	**16·3 ° Celsius**	**34·9%**

**Study Site: Berlin**		
**Date**	**Temperature** (Start-End)	**Humidity** (Start-End)
01.03.2021	21·7 - 28·5 ° Celsius	23% - 17%
02.03.2021	22·3 - 24·1 ° Celsius	23% - 20%
03.03.2021	23·8 - 26·2 ° Celsius	21% - 18%
04.03.2021	21·2 - 24·7 ° Celsius	23% - 19%
05·03·2021	23·8 - 23·4 ° Celsius	20% - 19%
09·03·2021	23·8 - 23·5 ° Celsius	19% - 18%
10·03·2021	23·3 – 25·9 ° Celsius	18% - 17%
11·03·2021	24·8 – 26·0 ° Celsius	21% - 21%
16·03·2021	22·2 – 25·2 ° Celsius	21% - 18%
17·03·2021	24·3 – 24·4 ° Celsius	19% - 19%
18·03·2021	23·5 – 22·6 ° Celsius	20% - 20%
22.04.2021	25·9 – 25·8 ° Celsius	18% - 17%
23.04.2021	24·8 – 24·8 ° Celsius	20% - 19%
24.04.2021	24·9 – 21·3 ° Celsius	23% - 22%
**Average**	**24·2 ° Celsius**	**19·9%**
